# A Data Science Approach for the Identification of Molecular Signatures of Aggressive Cancers

**DOI:** 10.3390/cancers14092325

**Published:** 2022-05-07

**Authors:** Adriano Barbosa-Silva, Milena Magalhães, Gilberto Ferreira da Silva, Fabricio Alves Barbosa da Silva, Flávia Raquel Gonçalves Carneiro, Nicolas Carels

**Affiliations:** 1 Center for Medical Statistics, Informatics and Intelligent Systems, Institute for Artificial Intelligence, Medical University of Vienna, 1090 Vienna, Austria; adriano.barbosa@ittm-solutions.com; 2 Centre for Translational Bioinformatics, William Harvey Research Institute, Queen Mary University of London, London E14NS, UK; 3 ITTM S.A.—Information Technology for Translational Medicine, Esch-sur-Alzette, 4354 Luxembourg, Luxembourg; 4 Plataforma de Modelagem de Sistemas Biológicos, Center for Technology Development in Health (CDTS), Oswaldo Cruz Foundation (FIOCRUZ), Rio de Janeiro 21040900, Brazil; milena.magalhaes@fiocruz.br (M.M.); gilberto.silva@fiocruz.br (G.F.d.S.); 5 Laboratório de Modelagem Computacional de Sistemas Biológicos, Scientific Computing Program, Oswaldo Cruz Foundation (FIOCRUZ), Rio de Janeiro 21040900, Brazil; fabricio.silva@fiocruz.br; 6 Center for Technology Development in Health (CDTS), Oswaldo Cruz Foundation (FIOCRUZ), Rio de Janeiro 21040900, Brazil; 7 Laboratório Interdisciplinar de Pesquisas Médicas, Instituto Oswaldo Cruz, Oswaldo Cruz Foundation (FIOCRUZ), Rio de Janeiro 21040900, Brazil; 8 Program of Immunology and Tumor Biology, Brazilian National Cancer Institute (INCA), Rio de Janeiro 20231050, Brazil

**Keywords:** cancer, aggressiveness, PCA, RFC, machine learning, interactome, RNA-seq, WNT pathways, prognostic genes

## Abstract

**Simple Summary:**

Traditionally, chemotherapy has been approached through one-size-fits-all strategies. However, personalized oncology would allow a rational approach to chemotherapies. Classically, cancer diagnosis and prognosis are performed through mutation mapping, but this genomic approach has an indirect relationship with the disease since it is based on the results of statistics accumulated over time. By contrast, a strategy based on gene expression would enable figuring out the actual disease phenotype and focusing on its specific molecular targets. In previous reports, we paved the way in that direction by successively showing that targeting up-regulated hubs are a suitable strategy to forward a tumor toward cell death and that the number of proteins to be targeted is typically between 3 and 10 according to tumor aggressiveness. In this report, we focused on the up-regulated genes of crucial cell signaling pathways, which are key hallmarks of unregulated cell division and apoptosis. By principal component analysis, we identified the genes that most explain the aggressiveness among cancer types. We also identified the genes that maximized the classification between aggressive and mild cancers using the random forest algorithm. Finally, by mapping these genes on the human interactome, we showed that they were close neighbors.

**Abstract:**

The main hallmarks of cancer include sustaining proliferative signaling and resisting cell death. We analyzed the genes of the WNT pathway and seven cross-linked pathways that may explain the differences in aggressiveness among cancer types. We divided six cancer types (liver, lung, stomach, kidney, prostate, and thyroid) into classes of high (H) and low (L) aggressiveness considering the TCGA data, and their correlations between Shannon entropy and 5-year overall survival (OS). Then, we used principal component analysis (PCA), a random forest classifier (RFC), and protein–protein interactions (PPI) to find the genes that correlated with aggressiveness. Using PCA, we found *GRB2*, *CTNNB1*, *SKP1*, *CSNK2A1*, *PRKDC*, *HDAC1*, *YWHAZ*, *YWHAB*, and *PSMD2*. Except for *PSMD2*, the RFC analysis showed a different list, which was *CAD*, *PSMD14*, *APH1A*, *PSMD2*, *SHC1*, *TMEFF2*, *PSMD11*, *H2AFZ*, *PSMB5*, and *NOTCH1*. Both methods use different algorithmic approaches and have different purposes, which explains the discrepancy between the two gene lists. The key genes of aggressiveness found by PCA were those that maximized the separation of H and L classes according to its third component, which represented 19% of the total variance. By contrast, RFC classified whether the RNA-seq of a tumor sample was of the H or L type. Interestingly, PPIs showed that the genes of PCA and RFC lists were connected neighbors in the PPI signaling network of WNT and cross-linked pathways.

## 1. Introduction

The worldwide estimate of people diagnosed with cancer was 18.1 million in 2018 [1], and it is predicted by the World Health Organization (WHO) to be 27 million new cases per year by 2030. The estimate is 70% higher 2040, with the consequence that cancer diseases will increase the economic pressure on nations, especially those with low incomes [2]. This threat has prompted the USA and Europe to fund huge projects to support cancer research.

Traditionally, cancer therapy has been dealt with through the one-size-fits-all approach. According to this approach, the chemotherapy protocol should fit every individual of a population. This concept is intrinsically imprecise, since it does not take into account the genetic peculiarities of each patient. Thus, a one-size-fits-all treatment approach does not work for everyone and may cause harmful side effects to many patients. By contrast, personalized oncology involves the tailoring of medical treatment to the (i) individual characteristics or symptoms and (ii) responses of a patient during all stages of care. Thanks to high-throughput techniques, the one-size-fits-all treatment is now undergoing a shift toward personalized oncology involving the identification of molecular pathways to predict both tumor biology and response to therapy.

The current precision oncology approach aims predominantly to induce apoptosis of cancer cells by blocking tumor-promoting signaling pathways [3]. Side-effects arising from the pleiotropic nature of genes have also been taken into account while addressing the pressing need of identifying new cancer drug targets, since downregulating a single pleiotropic gene affects a number of phenotypic traits in the same organism (see references in [4]. Similarly, drugs can also have pleiotropic targets, and their off-target activities may promote unintended biological effects [3]. In terms of their co-variation, direct links between human genetics and drug responses have been recorded in the Pharmacogenomics Knowledge Base (PharmGKB, https://www.pharmgkb.org/, accessed on 11 March 2022), which may help solve these issues [5]. An important part of precision oncology is in silico modeling, since it bridges the gap between molecular biology and the clinic to optimize patient therapy. Based on sequencing data, individual tumor drivers and related pathway interactions can be integrated into networks of key signaling cascades, which enable simplified mathematical modeling of systemic responses in tumors, such as apoptosis, proliferation, and resistance [6].

A recently proposed approach based on molecular phenotyping was the identification of the most relevant protein targets for specific therapeutic intervention in malignant breast cancer cell lines [7] based on the diagnosis of upregulated interactome hubs. This strategy combined protein–protein interactions (PPI) and RNA-seq data for inferring (i) the topology of the signaling network of upregulated genes in malignant cell lines and (ii) the most relevant protein targets therein (hubs). Hence, it has the benefit of allowing the association of a drug with the entropy of a target, and additionally, of ranking drugs according to their respective entropies with reference to their targets [8]. As a consequence: (i) A vertex (gene) with a high expression level is more influential than a vertex with a low expression level; (ii) a vertex with a high connectivity level (hub) is more influential than a vertex with a low connectivity level; (iii) a protein target must be expressed at a significantly higher level in tumor cells than in the cells used as a non-malignant reference to reduce harmful side effects to the patient after its inhibition. The use of the stroma as a control to measure the malignant differential expression via RNA-seq has been recognized as equivalent to using healthy tissues for this purpose [9]. It is worth mentioning that each combination of targets that most closely satisfied these conditions was found to be specific for its respective malignant cell line. These statements were validated in vitro in a breast cancer model by Tilli et al. [10].

The signaling network of a biological system is scale-free [11], which means that few proteins have high connectivity values and many proteins have low connectivity values. As proven mathematically by Barabási’s research group [12], the inhibition of proteins with high connectivity values has greater potential for signaling network disruption than randomly selected proteins [11]. This evidence was proven in silico by Conforte et al. [13] in the particular case of tumor signaling networks. In terms of systems biology, the inhibitory activity of a drug may be modeled by removing its corresponding protein target from the signaling network to which it belongs [8,13]. The impact of vertex removal from a network can be evaluated through the use of the Shannon entropy, which has been proposed as a measure and applied by many authors to determine a relationship between network complexity and tumor aggressiveness. The process outlined in Conforte et al. [13] was then automated, as described in Pires et al. [14].

The main hallmarks of cancer are the unregulated cell division and cell death resistance that lead to unlimited tumor growth, ending in the patient’s death. Many signaling pathways are involved in unregulated cell division. Nonetheless, the WNT pathway should be highlighted. WNT signaling controls a variety of biological processes, such as proliferation, apoptosis, cell motility, polarity, differentiation, and stem cell pluripotency. In addition, it can crosstalk with at least seven other pathways: EGF, FGF, HEDGEHOG, mTOR, NF-B, NOTCH, and TGF- [15,16,17]. The dysregulations of these pathways are also related to malignant transformation. Crosstalk can alter an individual pathway’s expected functions, resulting in a more complex and intricate network, which is difficult to predict. Therefore, we decided to analyze all these pathways’ components to better understand their contributions to cancer aggressiveness. In other words, we wanted to explore the components of the variance associated with the genes that might explain tumor aggressiveness. Our analysis showed that personalized therapy would (i) bring more benefits to the treatment of aggressive tumors (liver, lung, stomach) than to mild ones (kidney, prostate, thyroid) because the H class (highly aggressive) has a larger number of highly connected hubs than the L type (less aggressive) and (ii) shed light on which genes should be considered as negative indicators for 5-year overall survival (OS) by comparing tumors of mild and aggressive cancers using *principal component analysis* (PCA). We also took the opportunity to look for the genes that could be suitable as classifiers of aggressive and mild individual tumor samples using machine learning algorithms such as *random forest classifiers* (RFC). RFC has been successfully applied in cancer research to predict the primary sites of metastatic cancers [18], to detect meaningful somatic variations resulting from NGS analyses [19], and to perform the stratification of oropharyngeal cancer patients [20]. Both methods use different algorithmic approaches and have different purposes, which led us to investigate the protein–protein interactions of their gene lists by reference to the human interactome.

## 2. Materials and Methods

### 2.1. GDC Data

**Figure 1:**
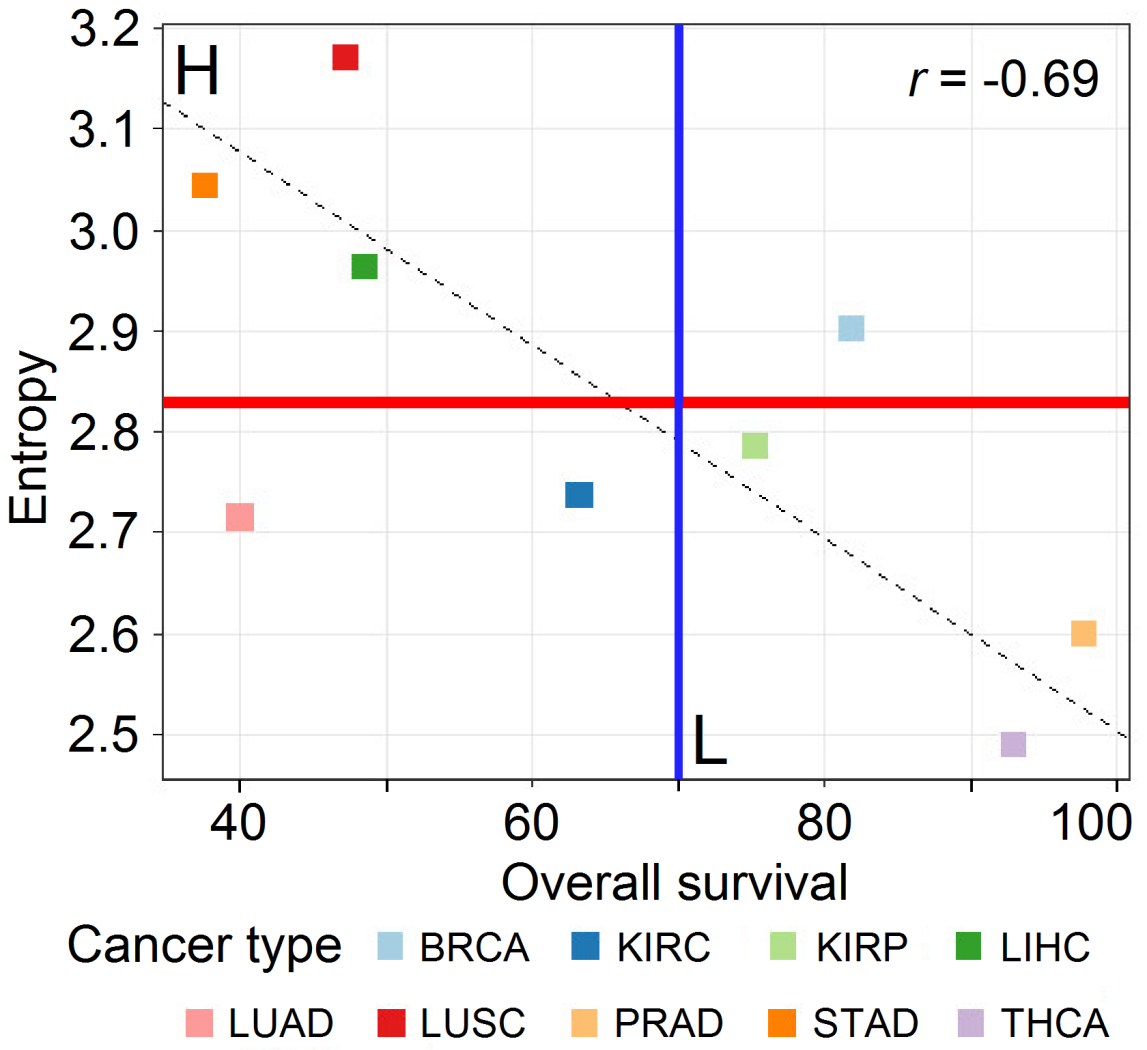
Correlation of subnetwork entropies vs. 5-year OS for *p* = 0.025 with GDC RPKMupper + Log2 (source: [14]).

The gene expression data were obtained as RNA-seq files from paired samples (control and tumor samples from the same patient) and downloaded from the GDC Data Portal (https://portal.gdc.cancer.gov/, accessed on 11 March 2022) in March 2020. The data selection followed two criteria: (i) for each cancer type, approximately 30 patients with paired samples were required to satisfy statistical significance, and (ii) the tumor samples had to be from solid tumors. The data we considered for this study were the same as in Conforte et al. [13] and Pires et al. [14]. They were from *stomach adenocarcinoma* (STAD, *n* = 27), *lung squamous cell carcinoma* (LUSC, *n* = 48), *liver hepatocellular carcinoma* (LIHC, *n* = 50), *kidney renal papillary cell carcinoma* (KIRP, *n* = 31), *thyroid cancer* (THCA, *n* = 56), and *prostate cancer* (PRAD, *n* = 48). By reference to Figure 1, drawn from Pires et al. [14], we considered the comparison among cancer types from two classes: highly aggressive (class H: STAD, LUSC, and LIHC) and less aggressive (class L: KIRP, THCA, and PRAD). To maximize the aggressiveness difference between cancer types, we excluded samples from BRCA, LUAD, and KIRC, since they could not be considered as typically belonging to H and L classes by reference to Figure 1. Table 1 displays the number of samples considered for each cancer class and tissue.

### 2.2. RNA-seq Processing

To process RNA-seq, we used the pipeline published by Pires et al. [14]. Briefly, we added BLASTn data to the pipeline [21]. After mapping the reads to the coding sequences from the protein of the 2017 version of the IntAct human interactome ([22], https://www.ebi.ac.uk/intact/, accessed on 11 March 2022), both the tumor reads and control reads (healthy or stroma) count files were normalized according to the size of their corresponding coding sequences. Then, the control RNA-seq was subtracted from the tumor’s one to calculate the differential expression. A step of logarithmic transformation was performed to reduce the bias introduced by genes with extreme expression values. The expression values of these genes varied a lot from one sample to another, since the variance increases with the average level of expression. The critical value of differential expression from each tumor–control pair was then calculated to identify the threshold above which genes were considered upregulated at a *p*-value of 0.025 given the 15,651 IntAct coding sequences.

### 2.3. Data Processing

We used R version 4 (https://www.r-project.org/, accessed on 11 March 2022) to perform the data analysis for this project. We opted to use R AnalyticFlow (https://r.analyticflow.com, version 3.1.8, accessed on 11 March 2022) as a framework where a major pipeline was established for the different steps reported here. A snapshot of the R analytical environment containing a list of dependency packages is available as Table S1. The processing flow was as follows:

*Data loading:* Data for six different cancer tissues have been used: three for the L class (KIRP, PRAD, and THCA: Table S2–S4 for clinical information) and three for the H one (LIHC, LUSC, and STAD: Table S5–S7, for clinical information). Every tissue dataset was composed of different TCGA samples files from the GDC portal, which listed its upregulated genes, UniProtKB identification number (corresponding proteins), and protein–protein connection number.

*Pathways:* We then inquired, for a set of eight cross-linked pathways (WNT, EGF, FGF, HEDGEHOG, mTOR, NF-B, NOTCH, and TGF-), the genes that were upregulated in cancer tissues samples. The gene members of each pathway were listed from the literature and extracted from KEGG ([23], https://www.genome.jp/kegg/, accessed on 11 March 2022) and Reactome ([24], https://reactome.org/, accessed on 11 March 2022) databases (Table S8). For those genes, we considered the number of connections (Table S9) and compared their normalized connections per cancer type (Table S10), as described in the *normalization* section.

*Normalization:* We normalized the number of counts for a given gene by the number of samples wherein such gene appeared to be upregulated in a given class (H or L), i.e., the number of times a gene was upregulated in samples of a given class was divided by the number of samples belonging to that class (e.g., ABL1 was upregulated 1 time in 125 samples belonging to class H, yielding a normalized count value of 0.008; see Table S9). The *normalized connections* for each gene were obtained by multiplying the normalized count by the number of connections considering all PPI, as it stands in the IntAct interactome (2017 version) (Table S9). This approach makes sense, since at a given moment, the interactions that proteins actually perform are expected to be proportional to their relative contributions to the whole PPIs. Thus, the normalized connection can be regarded as a PPI score.

*Variance analysis:* We used the Kruskal–Wallis test ([25]; R function: *kruskal.test*) to compare if the number of connections for each cancer within a pathway. Then, we used the Wilcoxon test ([26]; R function: *pairwise.wilcox.test*) for a pairwise comparison among cancers for the same pathway to find the relevant differences; we used the Bonferroni correction to adjust the *p*-value for multiple comparison (p.adjust.method = *bonf*).

*Filtering:* We used the normalized counts reported in Table S9 to summarize the mean and variance for each gene among classes H and L (Table S11) in order to select highly variant genes (variance > 0.005) for the principal component analysis [27]. However, only the genes that were upregulated in both categories (H and L) could have their standard deviation (SD) and variance compared, whereas genes only present in class H (148 genes) or class L (59 genes) could not (we report those genes in Table S12). We did not remove the genes that were only upregulated in just one category because they might be important severity indicators between cancers of different classes. Therefore, we attributed a *null* value (zero) to their *normalized counts*.

*Principal component analysis:* PCA can be used to identify the main axes of variance within a dataset and allows for easy data exploration to understand the key variables in the data and spot outliers [28]. In a recent application based on RNA expression profiles, a modified PCA approach (PCAUFE) was successfully used to identify genes that were critical for COVID-19 progression [29]. Dimensionality reduction techniques such as t-SNE [30] and UMAP [31] use loss functions as core methods, which make similar points to attract each other and push dissimilar points away from each other [32]. These features are useful when analyzing single-cell experiments [33], but not for identifying biological groups at a lower dimension, which was the case here.

To confirm the existence of two aggressiveness classes (H and L), we performed dimensionality reduction analysis using PCA with normalized connection data (*prcomp* function of R package stats version 3.6.2), (Table S10). All the descriptive features (highly variant genes) for the samples were reduced to two or three principal components (PCs) only, in order to separate classes into the bidimensional (2 PCs) or three-dimensional space (3 PCs).

*Hierarchical clustering:* In this section, we compared the efficiency of unsupervised hierarchical clustering (HC) with PCA for proper separation of H and L classes. For that purpose, we used the same set of genes as for the PCA analysis mentioned above and the *hclust* R function; the distance method adopted for the HC analysis was *manhattan*.

*Machine learning:* Given the cancer tissues division obtained by PCA into two classes (H and L) for a pool of samples from 6 different tissues, we used RFC to assess the power of machine learning into classifying individual TCGA samples as belonging to the highly aggressive (H) or less aggressive (L) classes, extracting therefore the most informative genes for such classification. The rationality behind this is that a sample coming from a less aggressive cancer type (e.g., KIRP) could display a pattern of gene expression that resembles those of a highly aggressive cancer (e.g., STAD), and it would be relevant to know its specific signature for personalized medicine purposes. We used the RFC algorithm [34] implemented in the *Random Forest* R package (https://cran.r-project.org/web/packages/randomForest/randomForest.pdf, accessed on 11 March 2022) for this purpose. As input, we used the presence or absence of upregulated genes (features) and their number of connections (values) in individual samples with parameters *mtry* = 8 (number of variables available for tree node splitting) and *ntree* = 50 (number of trees to grow). Thus, according to the *ntree* parameter, the success of the RFC classification was estimated using the out-of-bag error rate (OOB), where the classification trees are grown repeatedly (here 50 times) using 2/3 of the samples and tested against 1/3 of the remaining ones during the execution of the method; therefore, it was not necessary to split the input dataset into train and test subsets. Among the advantages to use RFC, one may cite its ability (i) to estimate variable importance [35], (ii) to work with missing data [36], (iii) to provide the highest classification accuracy [37], (iv) to handle big data with numerous variables [38], and (v) to cope with imbalance datasets [39].

*Network analysis:* We analyzed the protein–protein interactions of PCA and RFC selected genes by comparing them to the 2017 version of the InAct human interactome. We analyzed the extracted sub-interactome (Table S13) corresponding to these genes using Cytoscape ([40], https://cytoscape.org/, accessed on 11 March 2022).

## 3. Results

The authors van Wieringen and van der Vaart [41] found that, when considering transcriptome data, the entropy level of cancer samples is higher than that of normal samples. The same behavior was found among tumors: more advanced stages were characterized by higher entropy than the earlier ones [13,42,43,44]. Conforte et al. [13] confirmed the negative correlation between Shannon entropy and the 5-year OS described by Breitkreutz et al. [42] with TCGA data, which suggested that the signaling network of aggressive cancer is more branched than that of mild ones. On the other hand, the main hallmark of cancer is its uncontrolled cellular division. WNT and cross-linked pathways are known to be involved in this process [17,45]. We wanted to identify the genes within WNT and cross-linked pathways that may explain the differences in aggressiveness among cancer classes using Shannon entropy and 5-year OS. We investigated whether PCA and RFC would be suitable for this purpose.

### 3.1. Data Analysis

**Figure 2:**
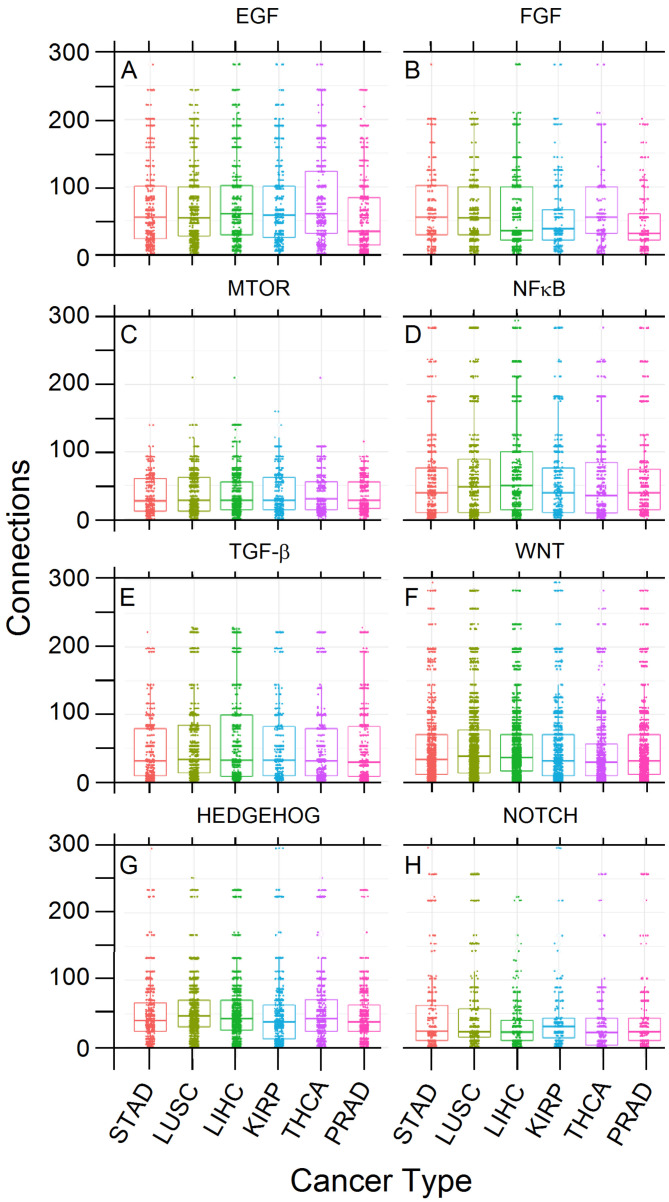
Frequencies (numbers) of upregulated genes’ connections per target pathway and cancer type. The plot is faceted per pathway and by Kruskal–Wallis *p*-values (**A**): 9.1 × 10${}^{-9}$, (**B**): 1.9 × 10${}^{-4}$, (**C**): 1.6 × 10${}^{-1}$, (**D**): 4.9 × 10${}^{-8}$, (**E**): 1.4 × 10${}^{-2}$, (**F**): 2.9 × 10${}^{-10}$, (**G**): 1.4 × 10${}^{-8}$, (**H**): 1.1 × 10${}^{-8}$. Each value indicates a group in which the average connection count is different from the global average within that facet.

After loading the samples of six cancer types (KIRP, PRAD, THCA, LIHC, LUSC, and STAD) into the R AnalyticFlow environment, we summarized the gene counts for each sample as a unique data frame, and we observed 9065 uniquely differentially expressed genes within those cancer types. Individual genes were annotated to their respective pathways, and we filtered those genes annotated as belonging to one of the target pathways (see Introduction). Finally, a total of 783 genes were selected for downstream analysis. From these 783 genes, (i) 724 were upregulated in the H class, (ii) 635 in the L class, (iii) 576 in both H and L classes, (iv) 59 only in L, and (v) 148 only in H. The number of connections for each of the selected genes and the comparison among pathways are displayed in Figure 2.

We performed a rank test using the Kruskal–Wallis method, which showed significant differences in the average numbers of connections within EGF (*p*-value = 9.06 × 10${}^{-9}$), FGF (*p*-value = 0.0001923), HEDGEHOG (*p*-value = 1.435 × 10${}^{-8}$), NF-B (*p*-value = 4.946 × 10${}^{-8}$), NOTCH (*p*-value = 1.135 × 10${}^{-8}$), TGF-β (*p*-value = 0.01458), and WNT (*p*-value = 2.923 × 10${}^{-10}$) pathways among the six cancer types. Only mTOR (*p*-value = 0.1645) did not show such significant differences (Table S14). In addition, a pairwise analysis using the Wilcoxon test within each pathway showed which cancers differed from another regarding the number of connections (Table S15); for example, KIRP differs from PRAD within the EGF pathway (*p*-value = 7.8 × 10${}^{-6}$).

Figure 2 shows that data are comparable and that classes are balanced, enabling further analysis without bias. However, some upregulated genes had remarkable distribution differences according to their relative frequency and connectivity among cancer types, which induced us to look at the PCA between tumors classified in two cancer classes according to their aggressiveness (H and L). Two examples of such distribution differences of two upregulated genes between H and L cancer classes are *PRICKLE1*, a WNT signaling component that contains six raw connections that was sampled more frequently in L tumors (49%) compared to H classes (2.5%) and *PSMC6*, a proteasomal subunit with 59 raw connections (hub) that was sampled more frequently in H tumors (40%) compared to class L (3%). These results paved the way for further comparisons between H and L cancer classes, as reported below.

**Figure 3:**
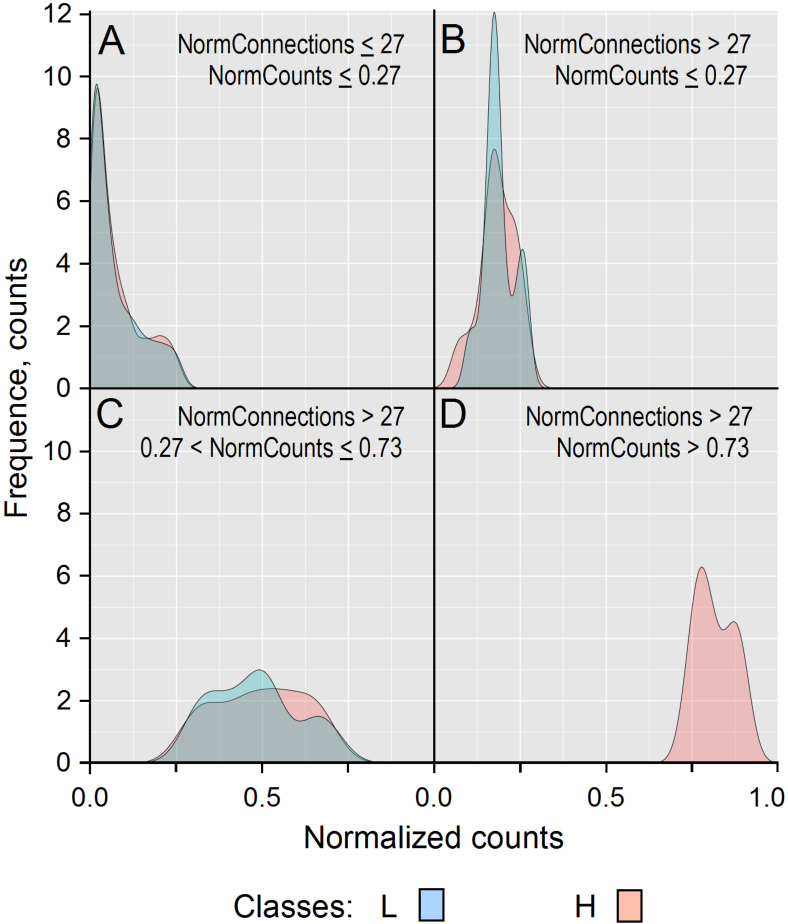
Frequencies (counts) of normalized gene counts (NormCounts) associated with genes of tumors from H and L cancer classes. From the 783 genes in this study, 609 were expressed in the H class and 559 in the L one. Panel (**A**) displays genes whose proteins had less than or equal to 27 normalized connections (NormConnections), and (**B**–**D**) those with more than 27 normalized connections. Considering proteins having less than 27 normalized connections (L = 509 and H = 531), frequency distributions were similar in L (blue) and H (red) classes for genes upregulated in less than 27% of tumor samples (**A**). Proteins having more than 27 normalized connections (L = 14 and H = 12) (BCD) are still present in a large proportion of the L class, even if they tended to be upregulated in more H class samples (**B**). The same trend was observed for genes upregulated in at least 27%, but no more than 73% of tumors of the L (*n* = 36) and H (*n* = 57) classes (**C**). This fact appeared clearly from panel (**D**), which shows that nine genes with at least 27 normalized connections were upregulated in at least 73% of the tumors from the H class, whereas no genes with such protein connection rates could be observed in the L class (**D**). These results show that considering the IntAct interactome as a reference, a protein can be considered as a hub when it has at least 27 normalized connections with its neighbors, and here we observed them in a larger proportion of aggressive cancers (class H), which shows that the WNT and cross-linked pathways are more branched in tumors of this class.

After normalization of the connection values obtained for the H and L classes, we compared their frequencies (see Methods for more details). Figure 3 shows that 9 genes with 27 normalized connections (NormConnections) and more than 0.73 normalized counts (NormCounts) were over-expressed only in cancer types from the H class, which meant that they could be considered as exclusive hubs. This number is the one that maximized the difference of connection distribution between L (blue) and H (red) classes.

Each panel of Figure 3 shows the smoothed distribution of normalized connections per gene and the normalized count (NormCounts) per gene across cancer classes. For instance, Figure 3D shows that among the genes whose proteins have more than 27 normalized connections with their neighbors, nine are present in more than 73% of tumors from the H class. These normalized values were used to filter highly variant genes for input of the PCA analysis as a matrix where the rows represent the cancer types and the columns the normalized connections for each gene (Table S10).

### 3.2. Principal Component Analysis

PCA is often used to reduce the dimensionality of a dataset, i.e., to reduce its variable number but keep most of its information content. By computing the covariance, PCA detects highly correlated variables, enabling it to minimize redundant information by combining them into principal components. Through PCA, the data were combined by successive maximization processes from the first to the last components. This organizing process allowed the neglect of the components with low information content. From a geometrical standpoint, principal components represent the directions of the data that explain a maximal amount of variance. Since the larger the dispersion along a line, the more the information it has, principal components can be assimilated to a system of new axes that provide the best angle to maximize the differences between data. A drawback of PCA is that it can only be indirectly interpreted since newly constructed variables are linear combinations of the initial ones [46]. Only the genes with variance of raw number of connections >0.005 in H and L (*n* = 450) and the ones present only in H (*n* = 148) or L (*n* = 59) classes from the six cancer types were used for the PCA analysis. From the 6 PCs yielded by the PCA analysis, the three first ones captured over 89% of the existing variance between samples of H and L classes (Table 2), with the third sharply distinguishing classes H and L.

**Figure 4:**
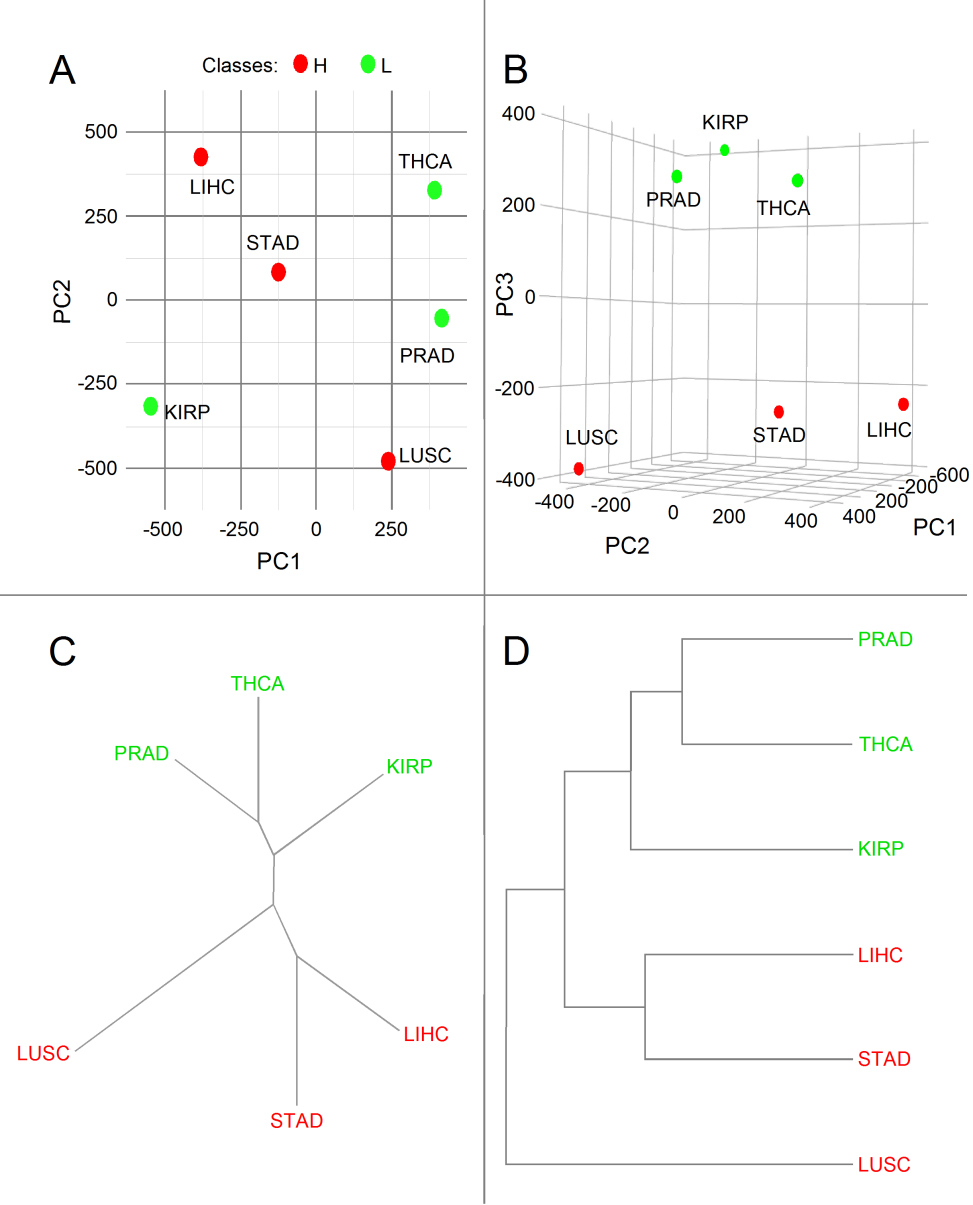
Bidimensional PCs (**A**) and three-dimensional PCs (**B**). *Principal component analysis* (PCA) representations of the variance of upregulated genes frequency considering the eight pathways and cancer type and dendrograms of hierarchical clustering of the PC3 components showing a clear division of cancer types into H (red) and L (green) classes. Unrooted (**C**) and rooted (**D**) representations.

The PCA associated with the upregulated genes known to be members of EGF, FGF, HEDGEHOG, mTOR, NF-B, NOTCH, TGF-, and WNT pathways gave the results of Figure 4.

We found a sharp separation of both L and H classes according to PC3 (Figure 4B). Since L and H classes were correlated with aggressiveness (Figure 1), we might conclude that PC3 was also related to this feature.

Given the complete separation between genes of H and L classes according to PC3, we corroborated the PCA results by *hierarchical clustering* (HC) analysis with the R function *hclust*. This HC analysis yielded the dendrogram displayed in Figure 4C,D, which shows a clear division of cancer types into H (red) and L (green) classes, with LUSC and KIRP being the most divergent members within each class.

The dendrogram represented in Figure 4C,D reinforces the results of Figure 1, since the entropies of PRAD and THCA are closer to one another than to KIRP. The entropies of STAD and LIHC are closer to one another than to LUSC.

Furthermore, Table S16 displays the percentage of contribution for each gene to the principal components PC1, PC2, and PC3.

Considering the genes (*n* = 10) that contribute at least 1% to PCA, a more careful analysis is given in Table 3, which reveals that they are all hubs due to their relatively high number of connections by reference to the transition zone (*n* = 27 normalized connections) between H and L classes (Figure 3).

As can be expected from hubs, they appear to be more frequent in the H class compared to the L one. Potentially, L genes may contribute to fewer PPI connections compared to H genes, according to the IntAct interactome. After multiplying relative frequencies (Norm. Cnt column) by connectivity (Norm. Cnx column) for H and L classes, the final scores were 1038.5 and 1953.1, for L and H classes, respectively—i.e., a score 47% larger for the H class compared to the L one (Table 3).

It is worth noting that genes from Table 3 are not necessarily the most frequent among tumors; rather, they are those whose distributions (presence/absence) according to their connectivity vary most among tumors. According to this consideration, GRB2 (86.4% of PC1) is the gene that contributes the most to the variation in WNT and cross-linked pathways (EGF and FGF) among tumors. The percentage of variance captured by PCA is given in relation to the total variance. Thus, if a given gene represents 50% of PC1 and PC1 represents 70% of the total variance, then this gene contributes 50% of 70% of the total variance. The same reasoning can be applied to the sum of the three main components. *CTNNB1* (EGF and WNT), *SKP1* (HEDGEHOG, NF-B, and WNT), *CSNK2A1* (NF-B and WNT), *PRKDC* (WNT), *HDAC1* (WNT), *YWHAZ* (WNT), *PSMD2* (WNT and HEDGEHOG), and *EGFR* (EGF) explain most of the variability among tumors (>80% of PC2). Finally, *CTNNB1* (EGF and WNT), *CSNK2A1* (NF-B and WNT), *PRKDC* (EGF, FGF, NF-B, and WNT), *YWHAZ* (WNT), *SKP1* (HEDGEHOG, NF-B, and WNT), *HDAC1* (NOTCH and WNT), *PSMD2* (HEDGEHOG and WNT), *YWHAB* (mTOR), and *GRB2* (EGF and FGF) are genes that most explain the difference of aggressiveness, by decreasing order of importance, between H and L classes (60% of PC3). Interestingly, let us note that the genes from the WNT pathways, cross-linked or not, that most contributed to PC3 accounted for 55.5%, i.e., 10.5% of the total variance, and that no genes from the TGF- pathway contributed significantly to PCs.

To verify whether the larger variance in *CTNNB1*, *CSNK2A1*, *PRKDC*, *YWHAZ*, *SKP1*, *HDAC1*, *PSMD2*, *YWHAB*, and *GRB2* associated with PC3 can be translated in a larger frequency in the H class compared to the L one, it is necessary to look at the original data. In the case of *PSMD2*, the answer is obvious since it is present only in the H class and can be considered a marker of aggressiveness. However, one can see in Table 3 that the other genes are between 1.4 and 3.8 times more frequent in the H class than in the L one, with (i) *SKP1*, *CSNK2A1*, *PRKDC*, and *YWHAB* differing by a factor larger than 2.5 and (ii) *CSNK2A1* and *PRKDC* differing by a factor larger than 3.0. Thus, *CTNNB1*, *CSNK2A1*, *PRKDC*, *YWHAZ*, *SKP1*, *HDAC1*, *PSMD2*, *YWHAB*, and *GRB2* are genes whose combination may, in principle, inform a physician about a poor prognostic in the context of a personalized gene diagnosis (see the “Assessing PCA and RFC Analyses” Section 3.4 below for a discussion).

### 3.3. Random Forest Classifier Analysis

*Decision Trees* are classification algorithms that proceed by successive steps of binary decisions according to different data features, which is equivalent to splitting data into groups by maximizing differences between them and the similarity among their respective members. RFC consists of a large number of individual decision trees that operate as an ensemble. Each individual tree in the random forest releases a prediction, and the one with the most votes is chosen as the predictive model. Splits in a classification tree are determined by minimizing a measure of misclassification known as *Gini impurity*. Gini impurity measures how often a randomly chosen record from the dataset used to train the model will be incorrectly labeled. Gini impurity reaches zero when all records in a node fall into a single category [47].

The genes that may be considered biomarkers of H and L classes predicted by RFC are given in Table 4 (see Table S17 for a complete gene list). The mean decrease in Gini is a measure of how much each variable contributes to the homogeneity of the nodes and leaves in the resulting random forest [47]. The higher the value of mean decrease accuracy or mean decrease Gini score, the higher the importance of the variable in the model. Thus, the genes with the largest Gini scores are the best H and L predictors. The values for H and L columns of Table 4 give the relative frequencies of genes in both H and L classes. One can see that except for *TMEFF2*, which are more frequent in the L class, the other genes are more frequent in the H class.

### 3.4. Assessing PCA and RFC Analyses

PCA enabled us to confirm the existence of two classes of aggressiveness based on the WNT and cross-linked pathways. Thus, PCA was valuable to figure out the structure of data. Regarding the use of PCA for finding prognostic markers, one has to note that PC3 only represents 19% of the total variance. Thus, if a gene such as *CTNNB1*, the gene of the WNT and cross-linked pathways that most contributes to PC3 or tumor aggressiveness, represents 14.02% of PC3 (Table 3), it, indeed, represents only 2.6% of the total variation. One can see that this frequency is very low, which suggests that *CTNNB1* cannot be considered alone a good prognostic marker in terms of clinical applications, taking into account the H class as a group. However, it can have prognostic value regarding specific types of tumors [48]. Since genes from Table 3 may account for a maximum of 19% of the total variance, it means that only a combination of them may inform a bad prognostic and not in every case because it depends on their personalized diagnosis that may vary from patient to patient. In addition, there may exist a confusion of the information due to aggressiveness and to other sources, as is the case for *GRB2*, for example. Considering *GRB2*, it contributes 1.49% to PC3 but contributes 86.39% to PC1. Thus, if one found a patient in which *GRB2* is upregulated, it would contribute 41% to the total variance and only 2.8% to aggressiveness. This indicates that *GRB2* is the gene that most varies among all the 6 tumor types analyzed. However, it is not a good indicator in predicting if a tumor belongs to the H or L class. This major dilution of PC3 information (aggressiveness) by other variance sources explains why RFC selects a different gene list to differentiate between H and L classes. The purpose of RFC is to maximize H and L classification independently of variance sources. However, the fact that we observed a separation between H and L classes for PC3 is a justification for classifying tumors according to aggressiveness using methods of artificial intelligence such as RFC.

By contrast to *CTNNB1*, the frequency of *PSMD2* in the H class is 42%, but it is 0% in the L class. This difference may justify why *PSMD2* is the only gene on the PC3 list considered a prognostic marker by RFC.

**Figure 5:**
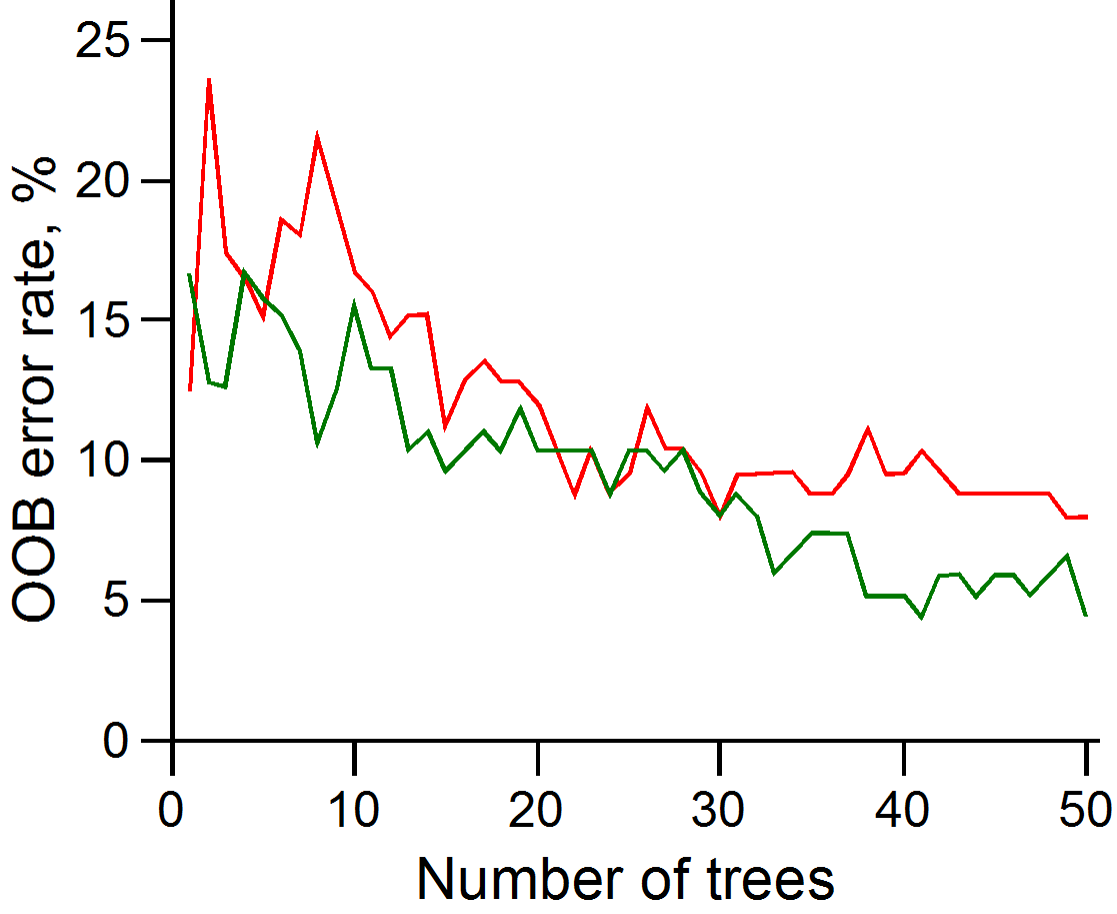
Evolution of the RFC error rate (OOB) with the increase in tree number (*ntree* parameter) for H (red) and L (green) classes.

With the RFC method, one uses the individual gene values as markers to classify which aggressiveness class a tumor sample is expected to belong to. This approach yielded results with error rates below 10% for the classification of both cancer classes H and L (0.08 and 0.04, respectively) as displayed in Table 5 and Figure 5.

### 3.5. PCA and RFC Gene Network

**Figure 6:**
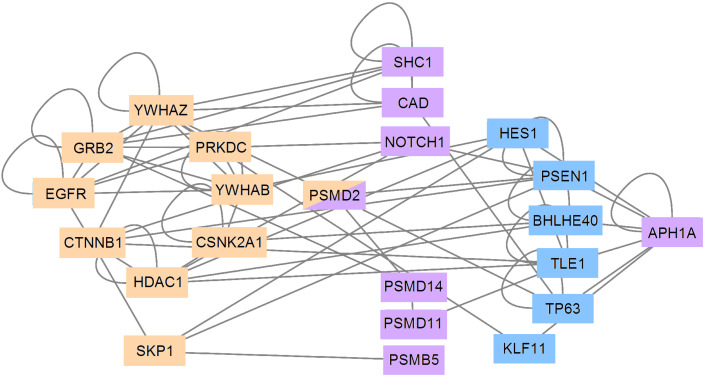
Interaction network of proteins from genes of Table 3 and Table 4. Table 3 (PCA) genes are represented on beige blocks and form a major component. Most genes of Table 4 (RFC) are represented on purple blocks and are from Table 3, except *APH1A*, *H2AFZ*, and *TMEFF2*. *H2AFZ* and *TMEFF2* were disconsidered here because they have low connectivity rates. *APH1A* is connected to the major network component through six intermediary proteins (blue) as appeared according to the IntAct interactome.

Except for *PRKDC*, all genes of Table 3 (PCA) are network hubs with large connection numbers in the IntAct human interactome and form a major component (Figure 6).

All genes of Table 4 (RFC) were directly connected to those of Table 3 except *APH1A*, *H2AFZ*, and *TMEFF2*. As shown in Table 4, these genes have low normalized connection levels and their probability of connecting to other vertices is rather small. Thus, *H2AFZ* and *TMEFF2* were disconsidered in the network of Figure 6. We found that *APH1A* connected with the major network component through six intermediary proteins. Interestingly, all of them, except for *PSEN1* (a transmembrane protein involved in Alzheimer’s disease), were transcription factors. In Figure 6, *PSEN1* (blue) is connected to (i) *APH1A* (purple), (ii) the two transcription factors HES1 (blue) and *BHLHE40* (blue), and (iii) NOTCH1 (beige), *PSMD2* (beige/purple), *CTNNB1* (beige), and *SKP1* (beige).

## 4. Discussion

At the moment, the most common strategy in oncology is to map mutations that promote suppressors and oncogenes [49,50]. Genomic alterations allow a diagnosis based on probabilistic data obtained with large patient cohorts. By contrast, the molecular phenotype, obtained by characterizing gene expression, portrays the cell or the genomic disease and points at proteins that should be targeted in the first instance to disrupt malignant phenotypes while affecting the healthy one the least possible. The phenotype approach also reflects which genes most malignant cells need to maintain in the tissue, given the selective constraints they may suffer [14].

The example of transcription factors associated with the glioma oncogene (GLI) demonstrates that multiple cancer-related signaling pathways converge on transcription factor families that may respond and generate positive feedback loops favoring malignant processes. Thus, these transcription factors represent molecular hubs whose inactivation controls the disease progression [51]. This case demonstrates through biological fact the concept of the Barabasi’s group (2016).

According to Albert et al. [11], complex communication networks display a surprising degree of robustness, which means that although key components regularly malfunction, local failures rarely lead to the loss of the global information content of a network. The stability of complex systems is often attributed to their heterogeneous and redundant wiring. It is precisely the rewiring through a few highly connected nodes that play a vital role in maintaining the network’s connectivity under random attack [12]. However, error tolerance comes at the high price of vulnerability when attacks are directed against hub vertices, with the consequence of quickly disarticulating the entire network into separated graphs. The networks that show these properties are called scale-free networks [11].

The relevance of inhibiting connection hubs has been proven mathematically by Barabási’s research group [12], and its benefit for cancer patients has been confirmed by Conforte et al. [13] through Shannon entropy analysis (see [52] for a review). The negative correlation found between the subnetwork entropy and the 5-year OS is in agreement with the results obtained later on from the modeling of basins of attraction in breast cancer with the Hopfield network [13].

Transcription factors belong to this group of connection hubs. Although several lines of evidence suggest beneficial effects resulting from GLI inhibition, one should consider that this pathway actively regulates many processes required for proper tissue regeneration and repair. Thus, it has been recognized that the inhibition of GLI signaling may eventually have negative benefits for patients (see refs in [51]). This is why choosing hub targets within the set of genes upregulated in the tumor compared to the stroma is essential. In this way, one may focus on the hubs (transcription factors or not) necessary to tumor maintenance while minimizing possible noxious effects on the patient.

It is argued that the interplay between several malignant pathways is one of the reasons why target-specific chemotherapies display low efficacy or even relapse, and it is the reason why multi-drug treatments need to be considered. With that respect, the negative correlation between subnetwork entropy and the 5-year OS, as described by Conforte et al. [13], suggests that the number of drugs within the treatment cocktail should be between 3 and 10 according to the tumor aggressiveness. Multi-drug therapies may need modeling because drugs can be administered to the patient in different combinations with different success rates [2,53].

The statement that more aggressive tumors have more complex signaling networks has a result of their higher average entropies compared to less aggressive ones is supported by the classical method of PCA applied to upregulated genes of WNT and cross-linked pathways, which showed a clear separation between H (aggressive cancers) and L (less aggressive) classes according to the PC3. Here, the point to be made is that the OS variable has not been informed to PCA. Thus, if the third component of PCA is able to separate tissues, it shows that tissues bring information to the system, and we know that this information is associated with tumor aggressiveness by reference to the negative correlation between entropy and OS. The variance is due to the fact that all tumors share most genes but with different frequencies that are specific to their tissues of origin. For instance, hubs are more frequent in tumors of the H group. This has the consequence that the number of alternative routes in the WNT and cross-linked pathways is more extensive and that their resistance potential to therapy is also more significant, which is a synonym of aggressiveness.

Thus, the classification in H and L classes based on the negative correlation between entropy and 5-year survival enabled us to identify the genes of the H class that have a worse prognosis compared to those of the L class. Among the genes revealed by PCA, *CTNNB1*, which encodes -catenin, is a critical effector of the canonical WNT signaling pathway. Its overexpression, protein stability, and nuclear translocation have been described in a wide range of solid tumors, especially in colorectal cancer (CRC), and hematological malignancies [54,55,56,57,58,59]. -catenin can have different functions depending on its subcellular localization. Both downregulated membranous -catenin and upregulated cytoplasmic and nuclear -catenin were associated with unfavorable prognosis in non-small cell lung cancer (NSCLC) [55]. Moreover, WNT/-catenin signaling plays a crucial role in epithelial-mesenchymal transition (EMT) in normal embryonic and tumor cells [58,60].

*CSNK2A1* encodes the catalytic subunit alpha of Casein Kinase 2 (CK2). CK2 is a constitutively active enzyme involved in transducing the signal of many different pathways [61], including mTOR, WNT, and NF-B. CK2 leads to antiapoptotic effects, potentiates multidrug resistance (MDR) phenotype, controls chaperones’ activity that stabilizes oncogenic proteins, and reduces tumor suppressor functions [61,62]. The overexpression of the CSNK2A1 subunit in different types of cancer makes this enzyme a strong target candidate for cancer treatment [63,64].

*PRKDC* encodes the DNA-dependent protein kinase catalytic subunit (DNA-PKcs), a critical DNA damage repair pathway component. DNA-PKcs is associated with chemotherapy resistance, tumor progression, metastasis, and poor survival in multiple human cancers. In addition, its expression was significantly associated with EMT signature, more advanced tumor grade, and faster progression [65,66,67,68]. Mechanistically, DNAPK interacts with LEF1 in prostate cancer. LEF1 is the primary transcription factor that mediates canonical WNT signaling [68]. On the other hand, DNA-PKcs overexpression regulates mTORC2-AKT activation and HIF-2 expression in renal cell carcinoma (RCC) cells [69] and has been involved in cell proliferation and oncogenic transformation by the stabilization of c-Myc oncoprotein via the AKT/GSK3 pathway in cancer cell lines [70].

*YWHAZ* and *YWHAB* belong to the highly conserved 14-3-3 protein family. The 14-3-3 family interacts with a wide range of cell signaling proteins through their phosphorylated sites [71] and the effect of 14-3-3 binding can vary depending on the target [72]. *YWHAZ*, also known as 14-3-3zeta or 14-3-3, is upregulated and correlated with poor patient outcome and metastasis in multiple human tumors [73,74]. Due to its extensive network interactions leading to pro-survival phenotypes, many studies suggest that this protein can potentially be targeted to sensitize cancer cells [72,75,76]. Interestingly, 14-3-3 binds to -catenin and other WNT/-catenin signaling proteins like GSK-3 and DVL-2 [73,77,78]. The 14-3-3/-catenin interaction decreases ubiquitinated -catenin, and therefore, its accumulation in the cytosol and nucleus in lung cancer cells. This interaction enhances cancer invasion by activating EMT-related genes and contributes to lung metastasis [73].

*YWHAB* (14-3-3) is also highly expressed in several tumors [79,80,81]. It promotes migration, invasion, and metastasis in hepatocellular carcinoma (HCC) [80,82] and is involved in cell proliferation of astrocytes and glioma cells [83]. By binding to GSK3-, 14-3-3 inhibits its activity, leading to -catenin stabilization and nuclear translocation [83]. Although there are still no 14-3-3 inhibitors in the clinic, several groups have been focusing on developing these molecules [75,76]. This report reinforces the need for the development of 14-3-3 inhibitors, especially for highly aggressive tumors.

*SKP1* (S-phase kinase-associated protein 1) is an adaptor protein of the Skp1/Cullin-1/F-box (SCF) E3 ligase protein complex. The SCF complex comprises unchanging components (Skp1, Rbx1, and Cullin 1) and the variable F-box proteins that confer substrate selectivity for ubiquitination. Many studies have correlated the overexpression of SCF components with malignant transformation and poor patient prognostic [84,85]. However, the role of SKP1 in tumorigenesis has only been investigated recently. SKP1 was overexpressed in NSCLC patient samples and CRC stem cells, being associated with a poor prognosis in both tumors [86,87]. On the other hand, it was suggested that SKP1 could have an anti-tumor effect in high-grade serous ovarian cancer (HGSOC) [88].

*HDAC1* belongs to the histone deacetylases (HDACs) family that deacetylates histone and non-histone proteins and plays fundamental roles in a broad range of cellular processes. The first non-histone protein shown to be deacetylated by HDAC1 was P53. Deacetylated p53 shows decreased p53-dependent transcriptional activation, less apoptosis, and reduced stability [89,90]. HDAC1 is overexpressed in many cancers and tumorigenic cell lines and promotes the proliferation of gastric, breast, and prostate cancer cells [91,92,93]. It is also involved in migration and invasion in breast and clear cell renal cell carcinomas (ccRCCs), respectively, [93,94].

*PSMD2* encodes a subunit of the 19S proteasomal regulatory complex. The proteasome is a multicatalytic protease that is crucial to protein degradation in eukaryotic cells. In general, the degradation occurs by the 26S proteasome (20S proteasome + 19S regulatory particle) and free 20S proteasome [95]. The proteasome degrades many proteins known to regulate oncogeneses, such as p53, p27, NF-B, TGF- receptor, and -catenin [96]. Therefore, it has a fundamental role in regulating essential pathways in cancer cells, such as tumor initiation and progression, and has been validated as an anticancer drug target [97]. *PSMD2* is overexpressed in different types of tumors and cancer cell lines, and it is correlated with poor prognosis in HCC, breast cancer, lung and gastric adenocarcinomas [98,99,100,101]. *PSMD2* is also associated with the metastatic phenotype in the lung cancer cell line NCI-H460-LNM35 and lymph node metastasis in breast cancer [99,100].

Finally, *GRB2* is a key adaptor protein that mediates the interaction between transmembrane receptors such as receptor tyrosine kinase (RTK) and non-receptor tyrosine kinase and downstream targets. Son of seven-less (SOS) is a crucial *GRB2* target that activates Ras and stimulates many pathways such as MAPK [102]. *GRB2* is critical to several tumorigenic processes. It is overexpressed in HCC tissues and cell lines, breast cancer, esophageal squamous cell carcinoma (ESSC), NSCLC, and is related to EMT, poor prognosis, and metastasis in several cancers [103,104,105]. Post-transcriptional regulation can also increase its oncogenic role [103,106,107].

Certainly, the presence of the hub genes identified by PCA in a tumor would most probably be a signal of poor prognosis and may serve as a risk indicator that would increase with the number of these genes being upregulated in a given tumor. However, as discussed above, the purpose of the genes identified by RFC is to classify tumors as belonging to H or L classes. Thus, the genes of the RFC list might be used as biomarkers of aggressiveness for a given tumor, while the genes pointed by PCA best explain the variance of aggressiveness between tumors, taken as a bulk, of H and L classes.

Amid genes that may classify tumors according to aggressiveness, RFC identified *PSMD2* (discussed previously), *PSMD11* (Proteasome 26S subunit, non-ATPase, 11), *PSMD14* (Proteasome 26S subunit, non-ATPase 14), which are components of the proteasomal 19S regulatory particle, and *PSMB5* (Proteasome 20S Subunit Beta 5), part of the 20S proteasome core. Interestingly, Tsvetkov et al. [108] showed that highly transformed cells exhibit increased dependency of the 26S proteasome amount and are extremely sensitive to its suppression. Aggressive and drug-resistant tumor cell lines like MDA-MB-231, Colo321, and HCT116 showed reduced proliferation after the knockdown of some 26 proteasome subunits. Accordingly, high levels of PSMD11 are associated with poorer overall survival [109] and were found in plasma-derived microparticles of pancreatic cancer patients with poor prognosis [110]. PSMB5 showed high expression in triple-negative breast cancer patients in addition to immuno-suppressive effects and oncogenic characteristics in several tumors [111,112]. Lastly, PSMD14 is a deubiquitinating enzyme and is overexpressed in several human cancers and predicts poor prognosis [107,113]. It targets important oncogenic proteins, stabilizing them, thereby contributing to tumorigenesis [107,113]. Interestingly, *GRB2*, identified in the PCA analysis, is one of its targets, reinforcing the crucial connections between the two lists of genes [107].

Another gene identified in RFC analysis was *CAD* (Carbamoyl-phosphate synthetase 2). It is a target of mTORC1 and plays a fundamental role in de novo pyrimidine biosynthesis and nucleic acid synthesis [114]. It is overexpressed or hyperactivated in many types of cancer and is related to poor survival, chemoresistance, and metabolic programming [115,116,117]. In addition, the proto-oncogene *MYC* positively regulates *CAD* expression [118], showing the critical role of *CAD* in tumorigenesis.

*APH1A* (anterior pharynx defective 1 homolog A) and NOTCH1, also found to be frequently upregulated genes in H class tumors, are components of the NOTCH signaling pathway, which when dysregulated, participates in a number of pathologies, including cancer and non-cancerous diseases [119].

*APH1A* was related to poor prognosis in pancreatic cancer [120]. It is a -secretase subunit, which is an intramembrane protease that cleaves a diversity of type 1 transmembrane substrates, including amyloid precursor protein (APP) and NOTCH receptors 1–4 [121,122].

NOTCH1 is a transmembrane receptor, and after its cleavage by -secretase, its intracellular portion transduces the signal [123]. The function of NOTCH1 in tumorigenesis is context-dependent, and it can act as an oncogene or tumor suppressor. In T-ALL, NOTCH1 is a very well-studied driving oncogene [119]. Moreover, NOTCH1 has a role in oncogenesis and metastasis in a wide range of solid tumors, including breast cancer and NSCLC [124,125]. Interestingly, Wieland et al. [126] showed that activated Notch1 is frequently found in endothelial cells of several human carcinomas promoting a senescence-like phenotype that facilitates tumor cell migration across the vessel wall and metastasis.

The *SHC1* gene is a central regulator of tyrosine kinase signaling and expresses three distinct isoforms, p46SHC, p52SHC, and p66SHC. The three isoforms contain a recognition site for the SH2 domain of the adaptor protein GRB2 [127]. p52SHC, in particular, is strongly related to Ras signaling and is required for progression and immune suppression of breast cancer [128,129]. On the other hand, the role of p66SHC in cancer development is ambiguous and depends on the cancer type [130].

*H2AFZ* encodes the H2A.Z.1 isoform of histone variant H2A.Z. Overexpression of *H2AFZ* was detected in many malignant cell lines and tumors [131,132,133,134]. It was related to poor prognosis in HCC [133], pancreatic ductal adenocarcinoma (PDAC) [134], and breast cancer [131]. Interestingly, in colon cancer cell lines, H2A.Z.1 expression is regulated by WNT signaling. Its depletion reduces cell proliferation and induces the expression of genes related to differentiated colorectal epithelial cells, indicating the importance of the WNT-H2A.Z.1 axis in intestinal homeostasis [135].

Interestingly, *TMEFF2* (transmembrane protein with egf-like and two follistatin-like domains 2), which encodes the single-pass transmembrane protein Tomoregulin-2, is described as a tumor suppressor protein. Its promoter and 5${}^{\prime }$-upstream CpG island are methylated, and it is downregulated in a wide variety of tumors [136,137,138]. However, the role of *TMEFF2* in prostate cancer is controversial, and several studies have shown opposite functions, oncogenic and tumor-suppressive, for this transmembrane protein [136,139,140]. In fact, the high number of reports showing opposing data on prostate cancer is intriguing. Our data in six types of cancers showed overexpression of *TMEFF2* in prostate tumors only, suggesting that this protein is not involved in tumorigenesis of highly aggressive cancers and that its expression could negatively control aggressiveness.

The fact that all PCA genes are connected suggests that they may be involved in similar processes, which is coherent with their contribution to PC3 (aggressiveness). It is interesting to note here that even if RFC tended to select different genes from PCA, most were directly (or indirectly as *APH1A*) connected to those resulting from the PCA analysis, which suggests some convergence between both methods. From a therapeutic standpoint, genes from Table 3 seem to be good theranostic targets since they are all hubs and interconnected [11,13]. Thus, their personalized diagnosis and inhibition would most likely lead to the effective disarticulation of the major component of the network, which is a molecular phenotype of aggressiveness. One may observe from Figure 6 that *YWHAZ* (eight edges), *YWHAB* (eight edges), *CTNNB1* (seven edges), *EGRF* (six edges), *GRB2* (six edges), and *PRKDC* (five edges) are the hubs that most contribute to the major component internal connectivity. *APH1A* is connected indirectly to the major PCA component and other RFC genes through six genes, of whom five are transcription factors. Interestingly, the only one that is not such an endpoint is *PSEN1*, which is a transmembrane protein [141] whose mutation may lead to Alzheimer’s disease [142].

## 5. Conclusions

This report showed that the distribution of upregulated genes of WNT and cross-linked pathways is correlated to tumor aggressiveness, with hub genes being more frequent in the aggressive class of cancer (H) than in the mild one (L). The variance among aggressive and mild cancer classes according to WNT and cross-linked pathways accounts for 19% of the total variance and is significant, since PCA can distinctly separate both cancer classes. From a prognostic standpoint and based on PCA, the genes that we found to be the most significant markers of malignant aggressiveness in WNT and cross-linked pathways were *CTNNB1*, *CSNK2A1*, *PRKDC*, *YWHAZ*, *SKP1*, *HDAC1*, *PSMD2*, *YWHAB*, and *GRB2*, in decreasing order of importance. By contrast, the genes found with RFC were *CAD*, *PSMD14*, *APH1A*, *PSMD2*, *SHC1*, *TMEFF2*, *PSMD11*, *H2AFZ*, *PSMB5*, and *NOTCH1*. Only *PSMD2* was found to be common to both lists. We believe the reason is that the purpose of PCA is to understand the structure of data according to the variance. By contrast, the primary purpose of RFC is classification. Thus, PCA shed light on the proportion of the total variance due to aggressiveness and the contributions of genes from WNT and cross-linked pathways to aggressiveness, whereas RFC focused on the genes that could classify tumors as aggressive or mild. The analysis of the network formed by PCA and RFC genes showed that both methods converged to some extent. However, PCA genes enabled focusing on the genes that most contribute to the molecular phenotype of aggressiveness, with the major hubs for theranostics being *GRB2*, *YWHAZ*, *EGFR*, *CTNNB1*, and *YWHAB*. The over-expression of these genes in a given tumor can also be a signal of a bad prognosis if associated with other parameters. Our study analyzed six tumors based on the work of Conforte et al. [13]. A more extensive analysis including other types of aggressive tumors, such as pancreatic cancer, and different signaling pathways, could lead to a better understanding of the differentiation between H and L classes. To the best of our knowledge, this was the first time that frequency and connection were analyzed as a combined variable. Our work highlighted the contributions of WNT and cross-linked pathways in the classification of tumor aggressiveness. This report should contribute to the improvement of rational approaches of personalized tumor therapy.

## Figures and Tables

**Table 1:** Summary of samples analyzed for classes H and L per cancer type.

Cancer Class	Cancer Tissue	Number of Samples	Total Samples per Class
H	STAD	27	125
	LUSC	48	
	LIHC	50	
L	KIRP	31	135
	THCA	56	
	PRAD	48	

**Table 2:** Proportions of variance explained by the first three PCA components.

	PC1	PC2	PC3
Standard deviation	523.245	355.632	334.339
Proportion of variance	0.475	0.219	0.194
Cumulative proportion	0.475	0.694	0.888

**Table 3:** Relative frequencies and connectivity of the upregulated genes that most contributed to PCA’s first, second, and third components.

	PCA						H Class			L Class		
Gene	PC1	PC2	PC3	% Tot Var ^1^	% Capt Var ^2^	Cnx ^3^	n ^4^	Norm Cnt ^5^	Norm Cnx ^6^	n	Norm Cnt	Norm Cnx
*GRB2*	86.39 ^7^	0.72	**1.49**	41.49	46.70	753	54	0.43	325.3	36	0.27	200.8
*CTNNB1*	0.01	41.47	14.02	11.83	13.31	444	81	0.65	287.7	62	0.46	203.9
*SKP1*	**3.03**	15.78	7.07	6.27	7.06	234	63	0.50	117.9	25	0.19	43.3
*CSNK2A1*	0.37	10.38	9.85	**4.36**	**4.91**	284	70	0.56	159.0	24	0.18	50.5
*PRKDC*	0.10	**4.50**	9.21	**2.82**	**3.17**	110	66	0.53	58.1	19	0.14	15.5
*HDAC1*	**1.97**	**4.09**	**4.17**	**2.64**	**2.98**	257	39	0.31	80.2	22	0.16	41.9
*YWHAZ*	0.72	**1.52**	7.38	**2.10**	**2.37**	522	109	0.87	455.2	67	0.50	259.1
*YWHAB*	**1.04**	0.96	**3.22**	**1.33**	**1.50**	310	83	0.66	205.8	33	0.24	75.78
*PSMD2*	0.14	**2.23**	**3.78**	**1.29**	**1.45**	132	53	0.42	56.0	NA ^8^	NA	NA
*EGFR*	0.18	**4.43**	0.35	**1.12**	**1.27**	464	56	0.45	207.9	43	0.32	147.8
Total	93.95	86.08	60.54	75.25	84.72	–	674	–	1953.1	331	–	1038.5

^1^ Percentage of total variance. ^2^ Percentage of captured variance. ^3^ Connection number. ^4^ Sample number. ^5^ Normalized counts. ^6^ Normalized connections. ^7^ The numbers for PC1, PC2, and PC3 represent the gene contributions (%) to their respective components. ^8^ NA is for: not available. The deep gray backgrounds represent relative frequencies larger than 5%, and the bold underlined numbers represent those between 1% and 5%.

**Table 4:** Gene contribution to the RFC classification of samples from the H and L classes ordered by decreasing importance according to their mean decrease in Gini score.

Gene	RFC Importance MeanDecreaseGini	Norm. Count. H	Norm. Count. L	Connections
*CAD*	2.64	0.39	0.04	60
*PSMD14*	2.47	0.74	0.20	63
*APH1A*	2.19	0.68	0.22	21
*PSMD2*	2.12	0.42	–	132
*SHC1*	2.08	0.71	0.16	102
*TMEFF2*	2.05	–	0.27	3
*PSMD11*	2.02	0.48	0.03	66
*H2AFZ*	1.98	0.84	0.27	7
*PSMB5*	1.92	0.68	0.25	38
*NOTCH1*	1.55	0.28	0.04	218

**Table 5:** RFC confusion matrix ^1^.

Class	H	L	Error Rate
H	115	10	0.080
L	6	129	0.044

^1^ Parameters: mtry = 8, ntree = 50, OOB estimate: 6.15%.

## Data Availability

The datasets supporting this report are available from https://portal.gdc.cancer.gov/ (accessed on 11 March 2022). The pipeline established for the different steps reported here is available from https://doi.org/10.5281/zenodo.5939429. Users interested by an interactive execution environment of the analytical pipeline can access a version deployed as an executable Jupyter Notebook file on Google Colab at: https://colab.research.google.com/drived/1QOp5D4TlD5nROVsQqDQMr84Nj1oeWw0a (accessed on 11 March 2022).

## Supplementary Materials

The following supporting information can be downloaded at: https://www.mdpi.com/article/10.3390/cancers14092325/s1, Table S1: List of R dependency packages; Table S2: Clinical data for Kidney Renal Papillary samples; Table S3: Clinical data for Prostate samples; Table S4: Clinical data for Thyroid samples; Table S5: Clinical data for Liver samples; Table S6: Clinical data for Lung Squamous Cell samples; Table S7: Clinical data for Stomach samples; Table S8: Gene members of each pathway listed from the literature and extracted from KEGG, Biocarta, and Reactome databases; Table S9: Gene members with their number of connections, frequency of overexpression, sample number, normalized counts, and normalized connections; Table S10: Genes with their normalized connections per type of cancer and aggressiveness; Table S11: Mean, Std. deviation, and variance of normalized counts from target target genes between H and L classes; Table S12: Genes that are specific to each cancer classe; Table S13: Sub interactome of proteins corresponding to the genes of Table 3 and Table 4 (except H2AFZ and TMEFF2 ) as well as Figure 6; Table S14: Kruskal-Wallis test by pathway; Table S15: Wilcoxon test of cancer types per pathway; Table S16: Percentage of contribution for up-regulated genes to the PCA’s first, second, and third components; Table S17: Gene importance as defined by RFC.
